# Factor structure of the patient health questionnaire-4 in adults with attention-deficit/hyperactivity disorder

**DOI:** 10.3389/fpsyt.2023.1176298

**Published:** 2023-07-14

**Authors:** Audun Havnen, Stian Lydersen, Arthur Mandahl, Mariela Loreto Lara-Cabrera

**Affiliations:** ^1^Department of Psychology, Norwegian University of Science and Technology, Trondheim, Norway; ^2^Nidaros Division of Psychiatry, Community Mental Health Centre, St. Olav’s University Hospital, Trondheim, Norway; ^3^Faculty of Medicine and Health Sciences, Department of Mental Health, Regional Centre for Child and Youth Mental Health and Child Welfare, Norwegian University of Science and Technology, Trondheim, Norway; ^4^Vårres Regional User-Led Center Central-Norway, Trondheim, Norway; ^5^Faculty of Medicine and Health Sciences, Department of Mental Health, Norwegian University of Science and Technology, Trondheim, Norway; ^6^Nidelv Division of Psychiatry, Community Mental Health Centre, St. Olav’s University Hospital, Trondheim, Norway

**Keywords:** anxiety, ADHD, depression, factor structure, mental illness, patient health questionnaire-4, psychometric properties

## Abstract

**Background:**

Persons with attention-deficit/hyperactivity disorder (ADHD) frequently experience symptoms of anxiety and depression. In this population, there is a need for validated brief self-report screening questionnaires to assess the severity of comorbid mental health problems. The Patient Health Questionnaire 4 (PHQ-4) is a self-report questionnaire that may contribute to this purpose as it can screen for both disorders efficiently. However, this will be the first study examining the factor structure of the PHQ-4 in samples of adults with ADHD, and also evaluating the validity of the Norwegian version of the PHQ-4.

**Objectives:**

The aim of the current cross-sectional study was to examine the factor structure and validity of the Norwegian version of the PHQ-4 in a sample of adults who reported having been diagnosed with ADHD.

**Methods:**

Of 496 invited, a total of 326 participants (66%) completed the PHQ-4, The World Health Organization Five Well-Being Index, the Oslo Social Support Scale and the 4-item Perceived Stress Scale electronically in a web-portal between the 9th and 30th of June 2020.

**Results:**

Confirmatory factor analysis of the PHQ-4 supported a two-factor structure [RMSEA = 0.038 (90% CI 0.000–0.159), CFI = 1.00, TLI = 0.999, SRMR = 0.004], consisting of a depression factor and an anxiety factor. Standardized factor loadings were 0.79 to 0.97. The PHQ-4 was negatively correlated with well-being and social support and positively correlated with perceived level of stress.

**Conclusion:**

This study indicates promising psychometric properties of the PHQ-4 as a measure of anxiety and depressive symptoms in adults with self-reported ADHD who are fluent in Norwegian. The questionnaire’s brevity makes it a valuable resource in research and clinical settings. However, more studies are needed to test the instrument in a clinical sample.

## 1. Introduction

Attention-deficit/hyperactivity disorder (1) has a prevalence of 2.8% in adults internationally (2). Persons with ADHD often struggle with psychiatric comorbidity (3–7), and adult patients frequently experience comorbid symptoms of anxiety and depression (8), to a larger degree than adults without ADHD (9). Although psychological (10) and pharmacological treatment (11) may be effective, still many patients are left untreated and better recognition and screening of adults with ADHD is needed. Even though general screening questionnaires and self-report measures of anxiety and depression have been developed, there is a need for validated questionnaires to measure comorbid psychiatric symptoms in adults with ADHD (12). Due to the core difficulties related to inattention, impulsivity and hyperactivity in ADHD (13), brief self-report measures are preferable. The development of reliable and validated brief screening questionnaires to assess the level of comorbid common mental health problems in persons diagnosed with ADHD is important both for improving future research as well as for use in clinical settings.

The 4-item Patient Health Questionnaire (PHQ-4) is a brief questionnaire (14, 15) which combines two items from the Patient Health Questionnaire 9 (16) and two items from the Generalized Anxiety Disorder Scale 7 (GAD-7) (17). The two items assessing depression are commonly referred to as PHQ-2 and the two items assessing anxiety are called GAD-2. Both of these two-item scales have been supported as valid measures of depression (18–22) and anxiety (23–25), respectively. Initially developed in the United States (15), the PHQ-4 has been translated and validated in multiple languages, including German (14), Spanish (26, 27), Greek (28), Korean (29) and Arabic (30). However, to date, a validated version of the PHQ-4 in Norwegian has not been available.

The PHQ-4 has been suggested as valuable for treatment management, due to the ability to provide rapid monitoring of treatment response and adherence (15). The psychometric properties of the PHQ-4 have been supported in mental health patients (15) and in the general population (14, 28, 31) and in a mounting number of recent studies with different populations [e.g., (32, 33)]. However, the PHQ-4 has not previously been validated in patients with ADHD. In studies measuring the PHQ-4’s construct validity, the PHQ-4 showed negative correlations with the World Health Organization-Five Well-Being Index (WHO-5) (34) and social support (35), and positive correlations with depression scales (32) and perceived stress (27) supporting divergent and convergent validity. It should be noted, however, that a study validating the Korean version of the PHQ-4 reported questionable discriminant validity (29) in a sample of psychiatric outpatients, which indicates the need to investigate the psychometric properties of the PHQ-4 in different populations. The reported internal consistency of the PHQ-4 has been acceptable to good in previous studies, with Cronbach’s coefficients ranging from 0.77 (33) to 0.86 (27). Nonetheless, the PHQ-4 has not yet been validated in adults with ADHD.

The PHQ-4 was originally conceptualized as a bidimensional measure of depression and anxiety (15). Confirmatory factor analysis (CFA) supports the proposed two-factor solution in primary care patients (15), in the general population (14, 32, 36), and in both infertile and pregnant individuals (34, 37). Although it has been questioned whether the PHQ-4 is suitable for assessing the severity of comorbid depression and anxiety in psychiatric clinic settings (29), the questionnaire is validated in primary care among patients with emotional disorders (15, 26).

In summary, brief self-report measures are preferable for adults with ADHD. The PHQ-4 may thus be a useful self-report measure to assess comorbid symptoms of depression and anxiety which are frequently reported in this population. However, the factor structure of the PHQ-4 has not been investigated in individuals with ADHD, and the validity of the Norwegian version of the PHQ-4 has not yet been explored. This study aims to examine, for the first time, the construct validity of the Norwegian version of the PHQ-4, including its factorial structure and internal consistency, in an adult ADHD sample.

## 2. Materials and methods

### 2.1. Study design and population

This anonymous cross-sectional survey was conducted between 9 June and 30 June 2020. Inclusion criteria were as follows: (1) to be 18 years and older; (2) to have proficiency in the Norwegian language; (3) to provide informed consent; and (4) to report to having been diagnosed with ADHD. We adhered to the taxonomy and methodology proposed by the Consensus-based Standards for the Selection of Health Measurements Instruments (COSMIN) (38) and the Strengthening the Reporting of Observational Studies in Epidemiology (STROBE) (39) when planning and conducting the study.

### 2.2. Recruitment and procedures

A total of 496 potential participants of an ADHD organization were contacted. All participants were invited via email by the study collaborators. In addition to describing the study’s aim and the use of the data, the emails contained an electronic link via Questback software to the questionnaire. Participants were presented with information about the study and were informed they could skip items and discontinue the survey at any time. By clicking “I agree,” the participants indicated that they had read and understood the information in the consent form, and that they agreed to participate in the research study. All participants reported that they had received a diagnosis of ADHD in the specialist mental health service, which currently uses the International statistical classification of diseases and related health problems (ICD-10) (40), but this was not confirmed through structural clinical interviews, and the time for the participants’ diagnosis was not recorded. The maximum time spent completing the scales was 12 min.

### 2.3. Measurements

#### 2.3.1. Patient health questionnaire 4

The Patient Health Questionnaire 4 (PHQ-4) (15) is a self-report measure of anxiety and depressive symptoms. The PHQ-4 has four items; two items from the PHQ-2 depression screener that assess core symptoms of major depressive disorder according to the Diagnostic and Statistical Manual of Mental Disorders, Fourth Edition (DSM-IV) (41) (“Over the last 2 weeks, how often have you been bothered by the following problems?”; ‘Feeling down, depressed, or hopeless’ and ‘Little interest or pleasure in doing things’). The two remaining items are from the GAD-2 anxiety screener and assess core symptoms of anxiety disorders (“Over the last 2 weeks, how often have you been bothered by the following problems?”; ‘Feeling nervous or anxious or on edge’ and ‘Not being able to stop or control worrying’).

The items are scored on a 0–3 Likert scale ranging from “not at all” (0) to “nearly every day” (3), with higher total score indicating higher symptom severity. The scores of the PHQ-2 and the GAD-2 items are added to calculate the composite PHQ-4 score, with a total score range between 0 and 12. In addition, in the present study participants also rated the degree of impairment due to anxiety and depressive symptoms by rating the question ‘If you checked off any problems, how difficult have these problems made it for you to do your work, take care of things at home, or get along with other people?” which was also scored on a 0–3 Likert scale.

#### 2.3.2. The World Health Organization five well-being index

The World Health Organization Five Well-Being Index (WHO-5) is a generic, self-reported scale that assesses perceptions of well-being. The scale includes five items, and higher scores represent higher perceived levels of well-being. The WHO-5 is a reliable and valid self-reporting questionnaire (42). Patients were asked to rate their agreement over the previous 2 weeks on each of the items rated on a 6-point scale. The five items are scored on a Likert scale ranging from “all of the time” (0) to “at no time” (5), with total score range between 0 and 25 (higher scoring indicates better well-being). The WHO-5 has previously been translated to Norwegian (43, 44).

Well-being is a key protective factor against the negative impact of depression and anxiety. In addition to measuring the construct of well-being, the WHO-5 also measures depressive symptoms, as it was originally developed to measure self-reported depression (42, 45). As such we expected that it would be a significant association between PHQ-4 and the WHO-5.

#### 2.3.3. The perceived stress scale 4

The Perceived Stress Scale 4 (PSS-4) (46) is a short questionnaire with four items that assesses the level of perceived stress. Each item is scored on a 0–4 Likert scale, with total score ranging from 0 to 16. Higher total score indicates higher degree of perceived stress. The PSS-4 is a reliable and valid questionnaire with good psychometric properties (46, 47). The scale has previously been translated to Norwegian (48).

Perceived stress is a common characteristic of the construct of anxiety. Therefore, consistent with previous studies (9, 27, 49) and based on the assumption that there are close relationships between anxiety and stress, we assumed there would be a significant association between PHQ-4 and PSS-4.

#### 2.3.4. The Oslo social support scale

The Oslo Social Support Scale (OSSS-3) (50, 51) is a 3-item questionnaire that measures social support. The total score ranges from 3 to 14, where higher scores equal stronger support. The total score may be used to indicate level of support: 3 to 8 indicates poor support; 9–11 indicates moderate support and 12–14 indicates strong support. Studies show that the OSSS-3 is a reliable and valid measure with sound psychometric properties (50, 52, 53). The Norwegian Directorate of Health has recommended to use this scale to measure the quality of life in Norwegian contexts (54).

Social support is a protective factor against psychological distress. Studies have shown that adequate social support is associated with lower symptom levels of anxiety and depression (35, 55, 56). Therefore, we expected PHQ-4 and OSSS-3 to be significantly associated.

### 2.4. Sample size

The target sample size (*n* = 120) was based on assuming a 10–12% dropout rate, with a significance level of 0.05 and power of 80%, based on general for recommendations for Cronbach’s *α* studies, and with an absolute minimum of 100 participants (14, 29).

### 2.5. Statistical analyses

**Figure 1:**
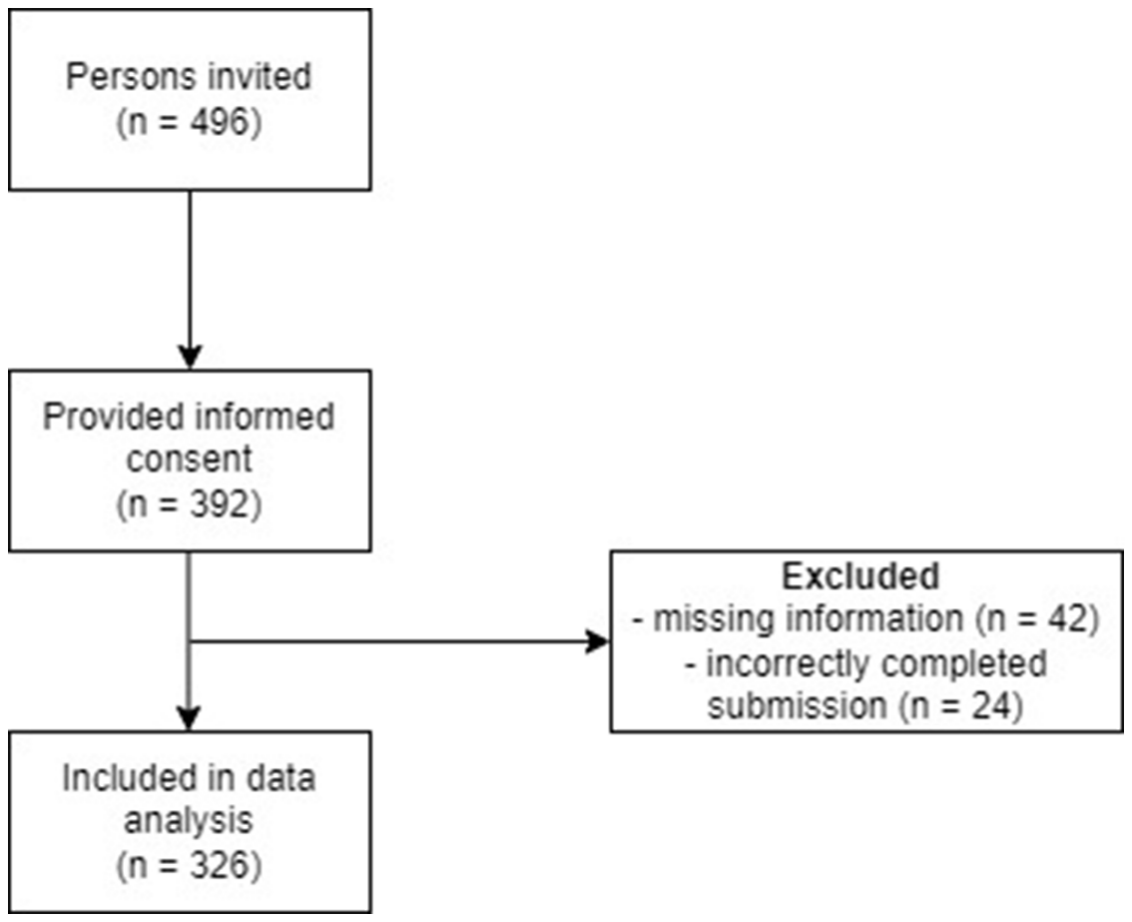
Flow chart of study participants.

A total of 496 adults who self-reported being diagnosed with ADHD were initially invited to participate in the study. Out of these, 392 individuals provided their consent to participate. However, due to missing information, 42 participants were excluded from the dataset. Additionally, 24 participants were removed due to incorrectly completing their submissions. Consequently, the final sample consisted of 326 participants, corresponding to 66% of the initially invited individuals (see flow chart in Figure 1). Cronbach’s *α* was calculated to assess internal consistency. Concurrent validity was investigated by calculating Spearman correlations between PHQ-4 and relevant measures, due to non-normally distributed data. These analyses were conducted using SPSS version 28.

Confirmatory Factor Analysis (CFA) was conducted to examine the fit of a one and two factor structure of the PHQ-4. Due to the ordinal nature of the Likert scale, the Weighted Least Squares Means and Variance adjusted (WLSMV) estimator was used. The model fit was evaluated according to multiple fit indices. Standardized Root Mean Square Residual (SRMR) (57) values below 0.08 indicate good fit. Root Mean Square Error of Approximation (RMSEA) (58) values from 0.00 to 0.05 are indicative of close fit, values between 0.05 and 0.08 indicate fair fit, values between 0.08 and 0.10 suggest mediocre fit, and values above 0.10 indicate poor fit. For the Comparative Fit Index (CFI) and the non-Normed Fit index (NNFI; aka Tucker–Lewis index; TLI) values greater than 0.95 indicate acceptable model fit (58). Multiple indicators, multiple causes (MIMIC) (59) modeling was used to test the effect of covariates in the model. Significant direct effects of a covariate on the factor indicate that the factor means are different at different levels of the covariate (59). In the MIMIC model, sex (women and men) and symptom severity (0–3 Likert scale) were included as covariates in the CFA model. CFA was conducted in Mplus version 8.8 (60).

## 3. Results

### 3.1. Participants’ characteristics

The sample consisted of 326 persons (226 women, 98 men, and two unknown). Age was reported in intervals. Lower education (primary or secondary school) was reported by 158 individuals, and 168 individuals reported higher education (college or university degree). Almost half the sample (*n* = 162) reported to be working full time, 30 were students, 41 were on sick leave, seven were on temporary lay-off, and 86 reported “other.” The majority (*n* = 198) reported to be married or living with a partner (Table 1).

**Table 1:** Demographic characteristics of the participants.

Characteristics	
Gender^a^	
Women, *n* (%)	226 (69.3)
Men, *n* (%)	98 (30.1)
Marital status, *n* (%)	
Married or cohabitant	198 (60.7)
Not married/cohabitant	93 (28.5)
Divorced/widow/widower	35 (10.7)
Age, *n* (%)	
18–24	23 (7.1)
25–29	26 (8.0)
30–34	55 (16.9)
35–39	52 (16.0)
40–44	57 (17.5)
45–49	50 (15.3)
50–54	38 (11.7)
55–59	14 (4.3)
60–64	7 (2.1)
65 or older	4 (1.2)
Education, *n* (%)	
Primary or secondary	158 (48.5)
College/university <4 years	107 (32.8)
College/university >4 years	61 (18.7)
Working status, *n* (%)	
Working	162 (49.7)
Student	30 (9.1)
Sick leave	41 (12.6)
Temporarily laid off	7 (2.1)
Other	86 (26.4)
PHQ-2, total score (0–6), mean (SD)	2.5 (1.7)
GAD-2, total score (0–6), mean (SD)	2.6 (1.7)
PHQ-4, total score (0–12), mean (SD)	5.0 (3.1)
PHQ-4 severity, *n* (%)	
None (0–2)	64 (19.6)
Mild (3–5)	142 (43.6)
Moderate (6–8)	71 (21.8)
Severe (9–12)	49 (15)
WHO-5, total score (0–25), mean (SD)	11.9 (5.1)
PSS-4, total score (0–16), mean (SD)	8.3 (1.6)
OSSS-3, total score (3–14), mean (SD)	8.6 (2.3)
OSSS-3 support level, *n* (%)	
Low (3–8)	158 (48.5)
Moderate (9–11)	129 (39.6)
Strong (12–14)	39 (12.0)

^a^Total count does not add up to *N* = 326, as two participants reported gender as “other.” PHQ-4, Patient Health Questionnaire 4. WHO-5, The World Health Organization Five Well-Being Index. PSS-4, Perceived Stress Scale 4. OSSS-3, Oslo Social Support Scale.

### 3.2. Mean differences and descriptive statistics

The frequency distributions of item responses are shown in Table 2. Participants tended to choose a value of 1 more frequently than other scores. GAD-2 item 2 (“Not being able to stop or control worrying”), and both PHQ-2 items showed floor effects. Women reported higher scores on PHQ-4 than men (Mean women *M_w_* = 5.3, SD = 3.2 versus Mean men *M_m_* = 4.4, *SD* = 3.0, *p* = 0.02).

**Table 2:** Internal reliability and item distribution.

Items						Frequency distribution *n* (%)				Item correlations		
	*M*	SD	Item-total correlation	*α*	*α* if item deleted	0	1	2	3	Item 2	Item 3	Item 4
PHQ-4 total	5.02	3.13		0.88								
Item 1: Feeling nervous	1.36	0.92	0.74		0.84	47 (14.4)	168 (51.5)	59 (18.1)	52 (16.0)	0.707*	0.539*	0.656*
Item 2: Control worry	1.21	0.95	0.75		0.84	75 (23.0)	151 (46.3)	56 (17.2)	44 (13.5)		0.538*	0.684*
Item 3: Little interest	1.25	0.89	0.66		0.87	54 (16.6)	179 (54.9)	50 (15.3)	43 (13.2)			0.665*
Item 4: Feeling down	1.20	0.91	0.79		0.82	70 (21.5)	160 (49.1)	57 (17.5)	39 (12.0)			

M, Mean. SD, Standard deviation. Item-total correlation, Corrected item-total correlation. *α*, Cronbach’s alpha. PHQ-4, Patient Health Questionnaire 4. *Significant corelation at *p* < 0.001. Correlations are Spearman’s rho.

### 3.3. Internal reliability

Internal reliability was evaluated using Cronbach’s *α*. Its value was 0.88, which indicates a high level of internal consistency (Table 2).

### 3.4. Validity

The PHQ-4 correlated negatively with well-being measured with the WHO-5 (*r* = −0.75, *p* < 0.001) and social support measured with the OSSS-3 (*r* = −0.38, *p* < 0.001), and positively with perceived level of stress measured with the PSS-4 (*r* = 0.31, *p* < 0.001). A summary of correlations between the measures is presented in Table 3.

**Table 3:** Correlations between PHQ-4 and other measures.

	M	SD	Min	Max	1	2	3	4	5
PHQ-4	5.0	3.1	0	12	0.917*	0.922*	−0.747*	0.313*	−0.380*
1. PHQ-2	2.5	1.7	0	6		0.711*	−0.743*	0.289*	−0.387*
2. GAD-2	2.6	1.7	0	6			−0.650*	0.302*	−0.297*
3. WHO-5	11.9	5.1	2	25				−0.232*	0.404*
4. PSS-4	8.3	1.6	0	15					−0.058
5. OSSS-3	8.6	2.3	3	14					

M, Mean. SD, Standard deviation. PHQ-2, Sum of first two items of Patient Health Questionnaire 9 items. GAD-2, Sum of first two items of Generalized Anxiety Disorder Scale 7 items. WHO-5, The World Health Organization Five Well-Being Index. PSS-4, Perceived Stress Scale 4. OSSS-3, Oslo Social Support Scale. *Significant correlation at *p* < 0.001. Correlations are Spearman’s rho.

### 3.5. Confirmatory factor analysis

**Figure 2:**
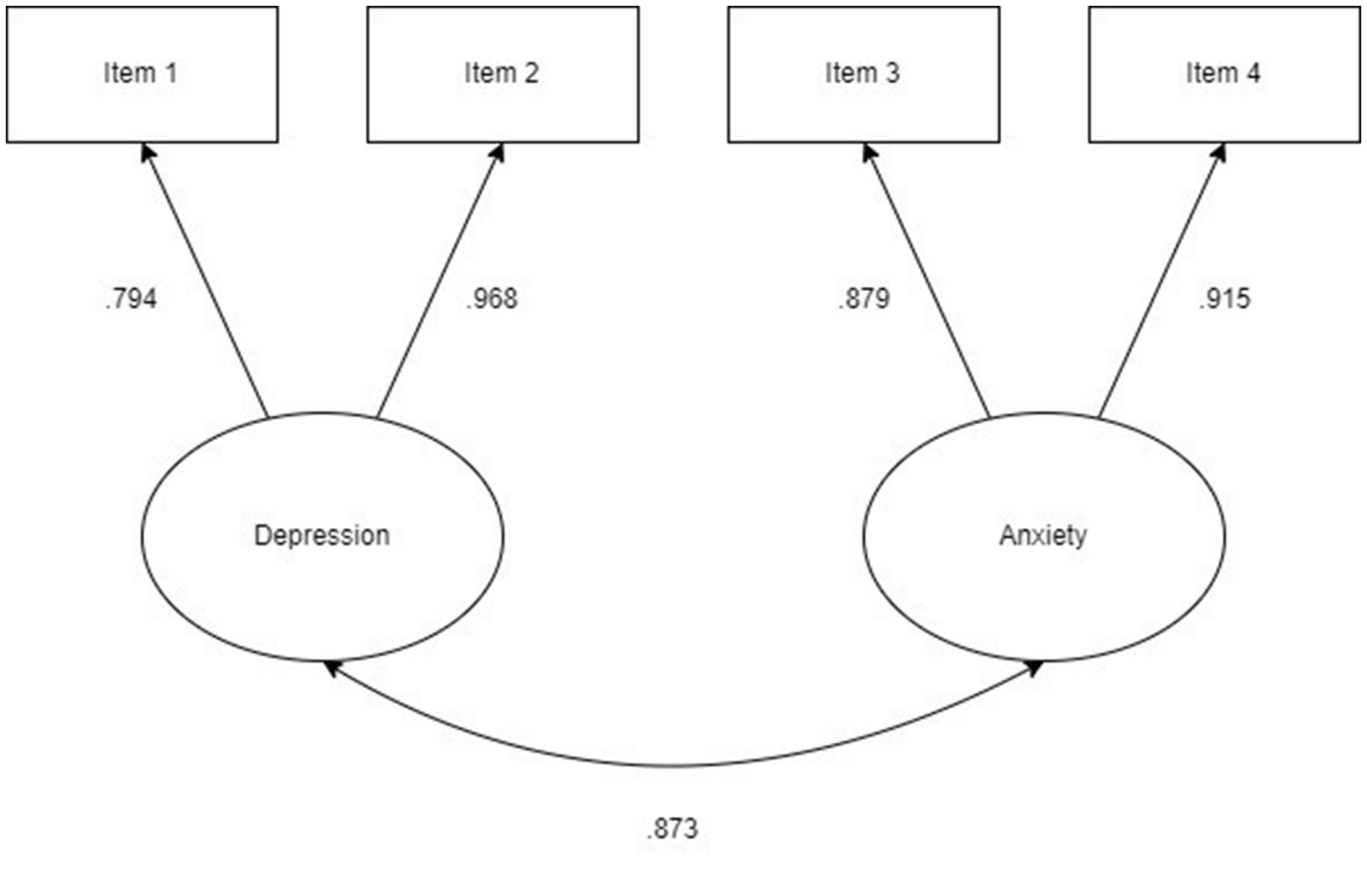
Confirmatory factor analysis of a two-factor model of PHQ-4. Standardized coefficients displayed. Arrow between factors represents correlation.

The CFA supported a two-factor solution for the PHQ-4. Fit indices supported a good model fit [RMSEA = 0.038 (90% CI 0.000–0.159), CFI = 1.00, TLI = 0.999, SRMR = 0.004] with 1 degree of freedom. Standardized factor loadings ranged from 0.79 to 0.97 (Figure 2). A one-factor structure was also tested, which showed less favorable fit indices than the two-factor structure [RMSEA = 0.231 (90% CI 0.169–0.299), CFI = 0.988, TLI = 0.963, SRMR = 0.026]. The fit indices of the one-and two-factor solutions are presented in Table 4.

**Table 4:** Goodness of fit for the two-factor and one-factor model of PHQ-4.

Model	*χ* ^2^	df	RMSEA [90% CI]	CFI	TLI	SRMR
PHQ-4 Two factors	1.464	1	0.038 [0.000, 0.159]	1.000	0.999	0.004
PHQ-4 One factor	36.558	2	0.231 [0.169, 0.299]	0.988	0.963	0.026

*χ*^2^,Chi-square statistic. df, Degrees of Freedom. RMSEA, Root Mean Square Error of Approximation. CFI, Comparative Fit Index. SRMR, Standardized Root Mean Square Residual. TLI, Tucker–Lewis index.

Sex and severity were included as covariates in the two-factor PHQ-4 model to test a MIMIC model, with the two factors *anxiety* and *depression* regressed on the covariates. The MIMIC model indicated a good model fit [RMSEA 0.059 (0.000–0.109), CFI = 0.996, TLI = 0.988, SRMR = 0.032]. The unstandardized estimate was significant for sex and anxiety (−0.270 *p* = 0.027) but not for sex and depression (0.115, *p* = 0.292). The negative estimate between sex and anxiety indicates that the mean score of men was 0.270 points lower than women on the anxiety factor. The unstandardized estimates between severity and anxiety (1.009, *p* < 0.001) and depression (0.964, *p* < 0.001) were significant, indicating that those who reported a higher level of impairment also scored higher on anxiety and depression, as expected.

## 4. Discussion

The aim of the current study was to investigate the factor structure and validity of the PHQ-4 in an adult sample of persons diagnosed with ADHD. A two-factor solution was found to be superior to a one-factor model. The psychometric properties of the PHQ-4 are promising, and our results supported the scale as a reliable, valid and brief tool for screening symptoms of anxiety and depression in adults with ADHD.

The CFA supported a two-factor solution consisting of an anxiety and a depression factor. The standardized factor loadings for item 2 and item 4 were high (*λ* = 0.968 and *λ* = 0.915, respectively), which suggest that these items may particularly important when screening for symptoms of depression and anxiety in adults with ADHD. The two-factor structure identified corroborates previous research which found the two-factor solution to be superior to a one-factor solution (32, 34, 36, 37), which is further in accordance with the factor structure proposed by the creators of the scale (15). One study found support for a one-factor model of PHQ-4 (61), but in contrast to our sample this study partly included both adolescents and young adults and the results are therefore not directly comparable to the present study. A study investigating the Korean version of the PHQ-4 in a sample of adult psychiatric outpatients also found a one-factor solution to be superior to a two-factor solution (29). However, the CFA in the latter study also showed poor model fit for the two-factor solution, and the sample did not include patients with ADHD, which means that these findings may not be comparable to the results of the present study. Why studies have found support both in favour of a one-and a two-factor solution is unclear, but the authors of the Korean validation study suggested that the high comorbidity between anxiety and depression in their sample may have influenced the results (29). In summary, the majority of previous studies appears to support a two-factor solution of the PHQ-4, and this is in line with the results of our study.

When testing whether men and women scored differently on the anxiety and depression subscales of the PHQ-4, results from the MIMIC model showed that the mean of the anxiety factor was higher for women than men. The sex difference in anxiety is in line with previous studies that have found women with ADHD to report higher levels of anxiety symptoms than men (62). However, the literature also typically finds women more likely to report higher levels of depression than men (63, 64), which was not found in the MIMIC model. Further, the MIMIC model showed significant estimates between severity and both anxiety and depression. This finding indicates that the means of the anxiety and depression factor were higher for those who reported a more severe degree of impairment, which was expected. This result shows that the PHQ-4 differentiates higher and lower levels of symptom severity.

The results showed high internal reliability, with a Cronbach’s alpha score of *α* = 0.88 which is in line with, or somewhat higher than, previous studies [e.g., (39)]. Reliability was not improved if any of the items were deleted. These results are encouraging and suggest that the PHQ-4 has good reliability as a measure of comorbid symptoms of emotional distress in adults with ADHD. Furthermore, the sample reported a mean PHQ-4 score of 5.0 (*SD* = 3.1), which is lower than the mean score of 7.2 (*SD* = 3.7) in a sample of adults with comorbid ADHD and problematic gambling (65). This may suggest that individuals who suffer from comorbid conditions in addition to ADHD may experience a higher severity of emotional symptoms compared to persons without comorbidity. However, as we did not assess comorbidity with structured diagnostic interviews, we have no information regarding the extent of comorbidity in the current sample. Using a PHQ-4 score equal to or above 3 as a cut-off to indicate a possible clinical level of symptoms, 39 and 36% scored above the cut-off for anxiety and depression, respectively, while 28% scored above both cut-off values. These results are similar to a German study that found that about 30% of adults who reported having ADHD scored above the cut-off value for anxiety and depression (66).

We investigated how well the PHQ-4 was associated with other measures. The results showed that the PHQ-4 correlated negatively with the WHO-5 and the OSSS-3 and positively with the PSS-4, with the strongest correlation between PHQ-4 and WHO-5. The WHO-5 has been supported as a valid screening tool for depression (45), and the analyses showed stronger correlations with the PHQ-2 than the GAD-2. The results of our study are thus in line with previous research (34, 43). The negative correlation between the PHQ-4 and OSSS-3 is in line with the well-established finding that social support is an important protector against level of anxiety and depression symptoms (55, 56). Furthermore, previous research has demonstrated a strong association between perceived level of stress and in particular anxiety (9), and perceived stress is found to mediate the relationship between ADHD symptoms and depression (49). The positive correlation between the PHQ-4 and the PSS-4 found in our study is thus expected based on previous research (43). Altogether, the PHQ-4 correlated in the expected direction with other validated measures which supports the validity of the PHQ-4 in adults with ADHD.

The study was conducted during the COVID-19 pandemic in Norway. This may have influenced the results, as studies have indicated a general increase in symptoms of anxiety and depression during the pandemic in the general public (67, 68) and among adults with ADHD (69). However, the mean PHQ-4 score resembled those of non-clinical community respondents in a Southeast Asian study conducted during the COVID-19 pandemic (36), who reported a mean score of 4.98 (*SD* = 3.22). The results of the current study thus did not indicate that there was a heightened level of symptom severity in adults with ADHD during the COVID-19 pandemic in Norway. It should also be noted that a Norwegian study did not find evidence of increased mental illness during the COVID-19 pandemic, but, on the contrary, reported a decrease in mental disorders from the pre-pandemic period to the beginning of the pandemic (70). Nevertheless, the results of the present study should be compared to future studies in order to investigate if the mid-pandemic sampling period potentially influenced the results.

### 4.1. Limitations

The findings of this study are limited by the use of self-report data. Participants reported having received a diagnosis of ADHD in the specialist mental health service. However, the time for diagnosis was not recorded and, in the present study, the diagnosis was not confirmed through structured clinical interviews. Moreover, we did not include validated measures of anxiety and depression to verify construct validity. Furthermore, the high factor loadings of items 2 and 4 of the PHQ-4 indicate that these questions are especially important when assessing depressive and anxiety symptoms in adults with ADHD. Future research needs to verify the findings in a sample of newly diagnosed ADHD-patients in a clinical setting to investigate if the results are replicable. The cross-sectional design did not allow us to investigate the test–retest reliability of the PHQ-4. Future studies should include an assessment at a follow-up to investigate longitudinal invariance. The use of electronic data collection may pose a challenge to less digitally literate persons, and it is unclear if the results of the current study are generalizable to the traditional pen-and-paper utilization of the PHQ-4. Furthermore, it is important to acknowledge that our sample is limited by an imbalance in gender representation, with women comprising 66 percent of the participants. This gender disparity warrants caution in generalizing our findings to the broader population. However, it is worth noting that this distribution is consistent with previous studies conducted in Norway with different outpatient populations (71, 72).

### 4.2. Conclusion

The preliminary evidence for the psychometric properties of the PHQ-4 is promising. The results showed high internal reliability, and a two-factor solution consisting of a depression factor and an anxiety factor showed acceptable model fit (RMSEA = 0.038, CFI = 1.00, TLI = 0.999, SRMR = 0.004), with standardized loadings between 0.79 and 0.97. Due to the well-known difficulties regarding attention and hyperactivity experienced by persons with ADHD, the PHQ-4 is a brief instrument that may be particularly useful in assessing comorbid psychological symptoms. However, future studies should further evaluate the PHQ-4 in newly diagnosed adult ADHD samples to investigate its psychometric properties in a clinical setting.

## Data availability statement

Data and materials are available on reasonable request.

## Ethics statement

The studies involving human participants were reviewed and approved by the Regional Committee for Medicine and Health Research Ethics in Mid-Norway. The patients/participants provided their written informed consent to participate in this study.

## Author contributions

ML-C and AM conceptualized the study. AH and SL performed the statistical analyses. AM contributed to the data collection, writing – reviewing, and editing. All authors contributed to the interpretation of the analyses and in critically reviewing – editing the manuscript, and read and approved the final manuscript.

## Funding

During the data collection ML-C was supported by a postdoctoral grant from The Liaison Committee for education, research and innovation in Central Norway (Samarbeidsorganet; project no. 90327500). The funding body had no role in the design of the study or the collection, analysis or interpretation of the data or in writing the manuscript.

## Conflict of interest

The authors declare that the research was conducted in the absence of any commercial or financial relationships that could be construed as a potential conflict of interest.

## Publisher’s note

All claims expressed in this article are solely those of the authors and do not necessarily represent those of their affiliated organizations, or those of the publisher, the editors and the reviewers. Any product that may be evaluated in this article, or claim that may be made by its manufacturer, is not guaranteed or endorsed by the publisher.
